# Objective differentiation of neonatal EEG background grades using detrended fluctuation analysis

**DOI:** 10.3389/fnhum.2015.00189

**Published:** 2015-04-23

**Authors:** Vladimir Matic, Perumpillichira Joseph Cherian, Ninah Koolen, Amir H. Ansari, Gunnar Naulaers, Paul Govaert, Sabine Van Huffel, Maarten De Vos, Sampsa Vanhatalo

**Affiliations:** ^1^Department of Electrical Engineering (ESAT), STADIUS Centre for Dynamical Systems, Signal Processing and Data Analytics, KU LeuvenLeuven, Belgium; ^2^iMinds Medical IT DepartmentLeuven, Belgium; ^3^Section of Clinical Neurophysiology, Department of Neurology, Erasmus MC, University Medical CenterRotterdam, The Netherlands; ^4^Neonatal Intensive Care Unit, University Hospital GasthuisbergLeuven, Belgium; ^5^Section of Neonatology, Department of Pediatrics, Erasmus MC-Sophia Children's Hospital, University Medical CenterRotterdam, Netherlands; ^6^Department of Engineering, Institute of Biomedical Engineering, University of OxfordOxford, UK; ^7^Department of Children's Clinical Neurophysiology, HUS Medical Imaging Center and Children's Hospital, Helsinki University Central Hospital and University of HelsinkiHelsinki, Finland

**Keywords:** asphyxia, detrended fluctuation analysis, multifractal, background EEG, brain monitoring

## Abstract

A quantitative and objective assessment of background electroencephalograph (EEG) in sick neonates remains an everyday clinical challenge. We studied whether long range temporal correlations quantified by detrended fluctuation analysis (DFA) could be used in the neonatal EEG to distinguish different grades of abnormality in the background EEG activity. Long-term EEG records of 34 neonates were collected after perinatal asphyxia, and their background was scored in 1 h epochs (8 h in each neonate) as mild, moderate or severe. We applied DFA on 15 min long, non-overlapping EEG epochs (*n* = 1088) filtered from 3 to 8 Hz. Our formal feasibility study suggested that DFA exponent can be reliably assessed in only part of the EEG epochs, and in only relatively short time scales (10–60 s), while it becomes ambiguous if longer time scales are considered. This prompted further exploration whether paradigm used for quantifying multifractal DFA (MF-DFA) could be applied in a more efficient way, and whether metrics from MF-DFA paradigm could yield useful benchmark with existing clinical EEG gradings. Comparison of MF-DFA metrics showed a significant difference between three visually assessed background EEG grades. MF-DFA parameters were also significantly correlated to interburst intervals quantified with our previously developed automated detector. Finally, we piloted a monitoring application of MF-DFA metrics and showed their evolution during patient recovery from asphyxia. Our exploratory study showed that neonatal EEG can be quantified using multifractal metrics, which might offer a suitable parameter to quantify the grade of EEG background, or to monitor changes in brain state that take place during long-term brain monitoring.

## Introduction

Development of neonatal care has led to an increasing interest in continuous brain monitoring for individually optimized neurological treatment. Scalp electroencephalography (EEG) is the most commonly used, non-invasive method in the continuous monitoring of brain function in the neonatal intensive care units (NICU). While neonatal EEG monitoring is becoming a standard of care in many NICUs, the interpretation of continuous EEG records in a 24/7 mode remains a global challenge, especially because of shortage of the special expertise required for an adequate visual reading (Boylan et al., 2010). One solution to this over the past decade has been the use of compressed displays, especially the amplitude integrated EEG (aEEG, a.k.a. CFM; De Vries and Hellström-Westas, 2005), which enables easier review of selected EEG features at bedside. However, it is now well recognized that aEEG trends are susceptible to artifacts that require special expertise in aEEG reading, and yet, the aEEG interpretation is qualitative and subjective.

The current main challenge in the EEG interpretation is to objectively and quantitatively characterize the spontaneous, ongoing brain activity, often called “background activity” in the EEG nomenclature. It has been shown that the EEG background is most informative when it comes to assessing acute state or predicting future outcome of the brain (Monod et al., 1972; Watanabe et al., 1999; Menache et al., 2002). Several background grading systems have been published over the years (Watanabe et al., 1999; Murray et al., 2009; Cherian et al., 2011; Walsh et al., 2011), and they combine visually (i.e., subjectively) observed EEG properties to yield one, holistic EEG grade. Perhaps due to the high interindividual variability and ambiguity in visual assessments, the clinically used EEG grading is remarkably rough. For instance, ranges of interburst intervals (IBI) between mild and moderate EEG grades go in 5 s increments from <5 to 5–10 s till >10 s, respectively (Murray et al., 2009; Cherian et al., 2011).

It is intuitively obvious, that so rough EEG grading cannot adequately reflect the time-varying physiological state of the brain, although it allows a technically straightforward translation of the criteria into automated classifiers. Intriguingly, classifiers based on these same criteria are not able to fully emulate the visual grading (Murray et al., 2009; Korotchikova et al., 2011; Stevenson et al., 2013), which indirectly calls for identification of novel EEG features with clinical relevance.

Recent work in basic neuroscience has provided ample evidence that many brain behaviors exhibit scale-free properties where dynamics of a given feature have no distinct spatial or temporal scale (Beggs and Plenz, 2003; Fransson et al., 2013; Iyer et al., 2014; Roberts et al., 2014). This was also recently shown to be the case for the EEG activity of full-term neonates that recover from perinatal asphyxia (Iyer et al., 2014; Roberts et al., 2014), one of the most common reasons for continuous EEG monitoring in the NICUs.

Scale-free dynamics in a complex system can give rise to self-similarity over temporal scales, i.e., long-range temporal correlations (LRTC), which may be assessed from the EEG using detrended fluctuation analysis (DFA; Peng et al., 1994; Hardstone et al., 2012). Recent work has shown that LRTC in brain function is significantly affected by various clinical conditions (Linkenkaer-Hansen et al., 2001; Parish et al., 2004; Stam et al., 2005; Monto et al., 2007). Compared to the conventional, visually defined EEG features, LRTC reaches beyond the visually perceived time scales, while it also integrates multiple time scales (from seconds to minutes) into one mathematically and conceptually transparent estimate. Moreover, DFA as a paradigm is very strongly supported by theoretical, experimental and clinical studies (for review, see Hardstone et al., 2012). In addition, a small number of neonates have been studied and long-range temporal correlations have been explored (Berthouze et al., 2010). Recent development of signal analysis methods has extended DFA to multifractal DFA (MF-DFA), which characterizes time series with multiple co-existent dynamic processes that may give rise to temporally local fluctuations including both extreme small and large magnitudes. Several studies have reported MF-DFA to offer additional insight in experimental and clinical neuroscience contexts (Kantelhardt et al., 2002; Zorick and Mandelkern, 2013). The neonatal EEG is, indeed, an example of signal with extreme, apparently erratic fluctuations in signal amplitudes, making it potentially suitable for MF-DFA paradigm as well. Notably, a recent study provided evidence of fractal behavior in time series of interburst intervals in preterm babies (Hartley et al., 2012).

The present study was set out to examine the possibility that neonatal EEG exhibits LRTC, which can be used as a feature to assess brain condition in a clinically relevant context. This entails answering the following questions: First, is it possible to assess reliable DFA exponents from the neonatal EEG at clinically relevant time scales from seconds to tens of minutes? Second, if DFA is suboptimal, can metrics from an existing MF-DFA paradigm reflect neonatal EEG in a meaningful way? Third, is it possible to use these measures to distinguish background grades of the neonatal EEG? The last question offers a benchmark to the existing analysis paradigms, while it is also directly relevant for development of novel features for automated background EEG classifiers.

## Materials and methods

The present study was designed to proceed sequentially. We first examined the mathematical and physiological feasibility of the DFA approach in neonatal EEG analysis, and we studied the DFA fluctuation plots for differences in our clinical populations (different EEG grades). After observing mathematical limitations with the DFA paradigm, the work was continued to test MF-DFA paradigm, and finally to benchmark MF-DFA metrics with clinical EEG grading.

### Dataset

We used altogether 1088 15 min epochs (total 272 h) from 34 neonates that were recorded for clinical reasons at Sophia Children's Hospital, Erasmus MC (Rotterdam, the Netherlands). These neonates were part of a larger cohort of term asphyxiated neonates who were monitored from March 2003 till August 2007. Other results from this cohort have been published previously (Deburchgraeve et al., 2008; Cherian et al., 2011; De Vos et al., 2011; Matic et al., 2014). The clinical inclusion criteria for EEG monitoring were: gestational age of 37–43 weeks with clinical features of encephalopathy, and having at least one of the following features of birth asphyxia: (a) arterial pH of umbilical cord blood ≤ 7.1, (b) Apgar score ≤5 at 5 min, and (c) high clinical suspicion (like fetal distress, umbilical cord prolapse, difficult labor, or a history of convulsions). Exclusion criteria were: congenital cardiac abnormalities or other multiple anomalies, inborn errors of metabolism, and constantly isoelectric (<5 μ V) EEG activity. The study had the approval of the Erasmus MC Medical Ethical Review Board. Informed consent was obtained from the parents/guardians prior to the onset of the registration. The EEG recordings started 2–48 (median 19) h post-partum. The median duration of EEG was 31 (range 20–96) h. Seizures were recorded in 18 patients, however this clinical information was not considered in the later analyses further than confirming that distribution of DFA/MF-DFA metrics was not statistically significantly different between babies with vs. without seizures (calculated separately for each background category).

#### EEG sampling

The EEG was recorded with NicOne EEG recorder (Cardinal Healthcare, Madison, WI, USA) at sampling frequency of 256 Hz and our current data analysis was performed using bipolar montage with 12 derivations. Four epochs of 2 h continuous EEG recordings were selected per neonate with a minimal distance between them longer than 4 h.

### Feasibility of DFA in the neonatal EEG

**Figure 1:**
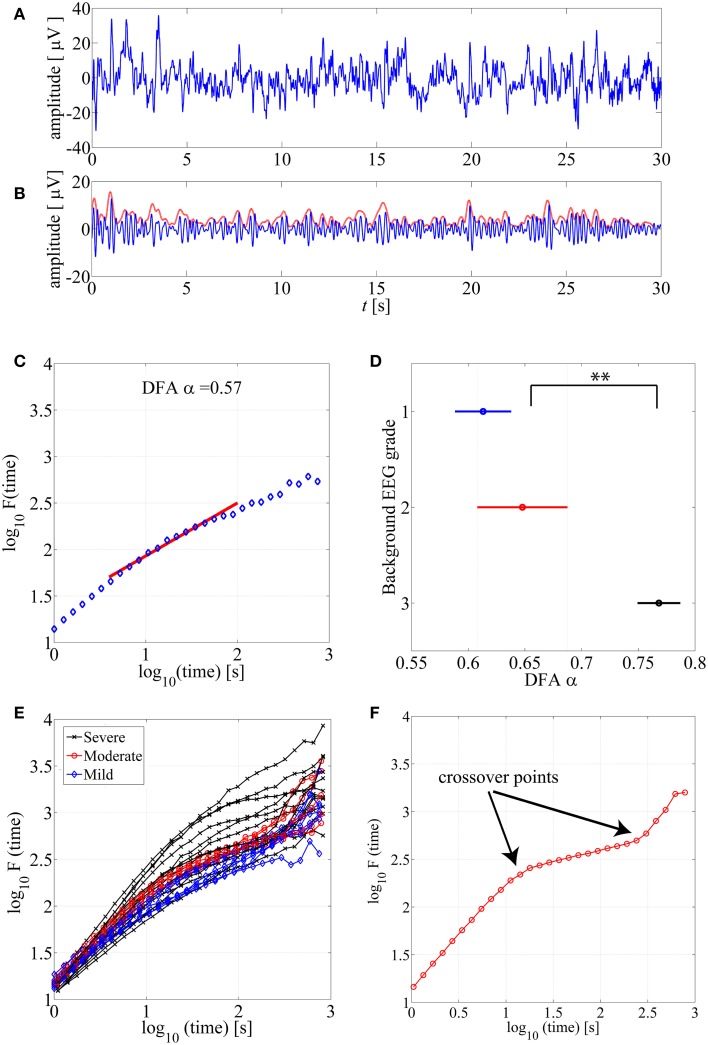
**(A)** An example of normal neonatal EEG signal with continuous background pattern. **(B)** The signal is band-pass filtered between 3–8 Hz (blue), and the instantaneous amplitude envelope (red) is calculated using Hilbert transform. **(C)** The DFA fluctuation function is presented along the Y axis, whereas the size of the windows analyzed in the DFA method are displayed in the X axis. The red line presents an example of linear fluctuation function that is here fitted for the time scale 10–60 s to obtain its slope, the DFA scaling exponent α. **(D)** DFA fluctuation functions are plotted for 15 min EEG epochs from different background EEG abnormalities (Mild-blue, Moderate-red, Severe-black). Statistically significant differences can be observed between different background EEG classes (^**^p < 0.01). This figure only shows examples of such EEG epochs where linear fit (*R*^2^ > 0.97) was confirmed. **(E)** Comparison of scaling exponent α between different background EEG grades shows a significant difference between grades 2 and 3. **(F)** An example of the DFA fluctuation function (moderate EEG grade) where clear existence of the crossover points are illustrated.

Detrended Fluctuation Analysis (DFA), originally introduced by Peng et al. (1994), is a widely applied method to quantify LRTC in various biological time series that exhibit self-similarity over multiple time scales. In the context of EEG analysis, DFA is typically computed by measuring amplitude dynamics of selected frequency bands (cf. Hardstone et al., 2012). In the first step, after band-specific amplitude extraction, the DFA procedure splits the signal into time windows (time scale—*s*), detrends the EEG segment, and computes the variance of amplitude fluctuations. In the second step, a DFA plot is generated by plotting the average signal variance per each window size on log-log coordinates (time scale—*s*, Fluctuation function—*F* in Figures 1C,E). Finally, a linear fit of this fluctuation function gives the DFA output, the scaling exponent α (cf. Appendix A; Hardstone et al., 2012).

Use of DFA paradigm requires defining several parameters so that they comply with the *a priori* known characteristics of the given biological signal. These include definitions of (1) the frequency band which is used for the signal envelope extraction (2) the epoch lengths, *time scales*, that are used to calculate the fluctuation function as well as (3) the range of time windows over which the slope of the linear fit (exponent α) is calculated (see Figure 1C).

#### Epoch length

We analyzed data epochs of 300, 600, 900, and 3600 s duration, but the final analysis was then carried out using 900 s (15 min) epochs. The choice was to strike a practical compromise between obtaining enough data for a reliable DFA plot, yet having likely a consistent background state in the given window. Our clinical background grading was performed initially in 15 min epochs, hence 900 s was practical for benchmarking purposes.

#### Frequency band

Neonatal EEG, especially in the acute state after asphyxic injury as in our data series, is dominated by intermittent bursting activity that consists of oscillations mostly at about 0.1–10 Hz. The lowest frequencies may be unpredictably contaminated by movement and other artifacts, so they were filtered out using a FIR highpass filter at 3 Hz. We chose to use a lowpass FIR filter at 8 Hz to maximally limit our analysis to the previously described 3–8 Hz band (Palmu et al., 2010; Tokariev et al., 2012; Omidvarnia et al., 2014). However, we also examined other frequency bands (1–3 and 1–5 Hz), and we got qualitatively comparable findings (data not shown).

#### Time scale (DFA window)

Feasibility of the DFA method requires that the signal does exhibit genuine LRTC, which in an ideal case could be seen over all time scales up to the limits of the recording time. The shortest time scale is defined by the need to include multiple fluctuation cycles of interest to really assess its temporal correlations (cf. Hardstone et al., 2012). In the present context, the amplitude fluctuations in the early recovering neonatal EEG consist of intermittent bursting at multi-second scale. The selected frequency band (3–8 Hz) gives slowest fluctuations with a duration of around 1 s (Tokariev et al., 2012), which suggested to use the lowest time scale of 10 s. The upper range can be physiologically limited by the spontaneous changes in the brain state over time, especially vigilance state cycling that occurs 1–3 times per hour in the neonatal brain (Stevenson et al., 2014). However, the upper limit of feasible scales can also be reasoned mathematically, since estimation of the DFA exponent α is based on linear fitting in the DFA plot over the given time scale (see below; Figure 1C).

Next, we continued quantitatively by analysing how well the linear fitting suits to different ranges of windows (5–60, 10–60, 10–100, and 10–300 s) in the fluctuation functions. Two independent techniques were used: The first method was developed by Botcharova et al. (2013) based on the maximum likelihood approach that compares a set of alternative models: linear, polynomial, root, exponential, logarithmic, and spline functions. This paradigm identifies the best fit model when the involved number of parameters is minimal and over-fitting is penalized. The second method was less strict, and adopted from prior studies (e.g., Linkenkaer-Hansen et al., 2001), based on computing the standard linear regression fit, *R*^2.^, and using a predefined threshold of 0.97 to indicate acceptance of the epochs for DFA analysis. More details about both results are shown in the Supplemental Data and they confirm that results obtained with the maximum likelihood based approach are valid.

### Feasibility of MF-DFA in the neonatal EEG

**Figure 2:**
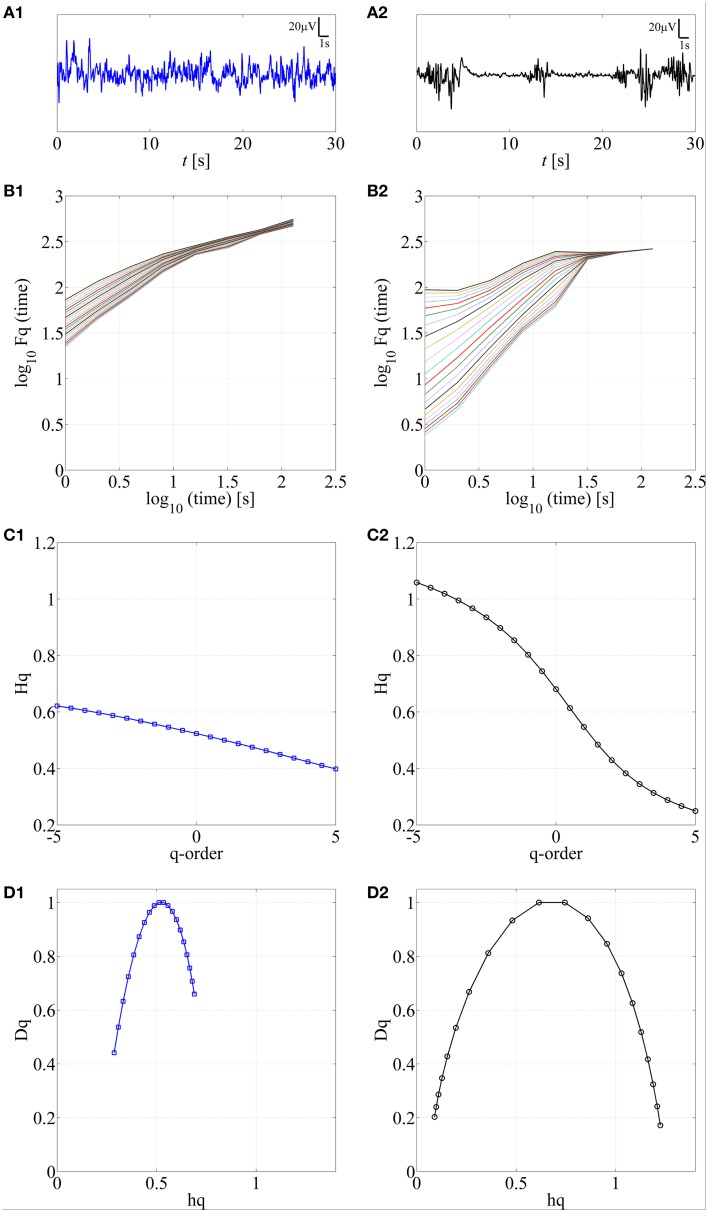
**Application of MF-DFA on two different neonatal EEG signals, continuous (A1) and burst-suppression (A2), respectively**. In **B1,B2**, the *q*-order fluctuation function, *F_q_*, is shown for two processes. **C1,C2** *q-*order Hurst exponent, *H_q_*, is obtained as the linear fitting coefficient of the *F_q_* fluctuation lines from **B1,B2**. Wider range of *H_q_* values is associated with the larger multifractal dimension. In contrast, a constant line is expected for monofractal time series. **D1,D2**. Multifractal spectra are displayed for continuous and burst-suppression neonatal EEG periods. Longer tails and wider width between the tails in **D2** compared to **D1** are associated with a higher degree of multifractality.

**Figure 3:**
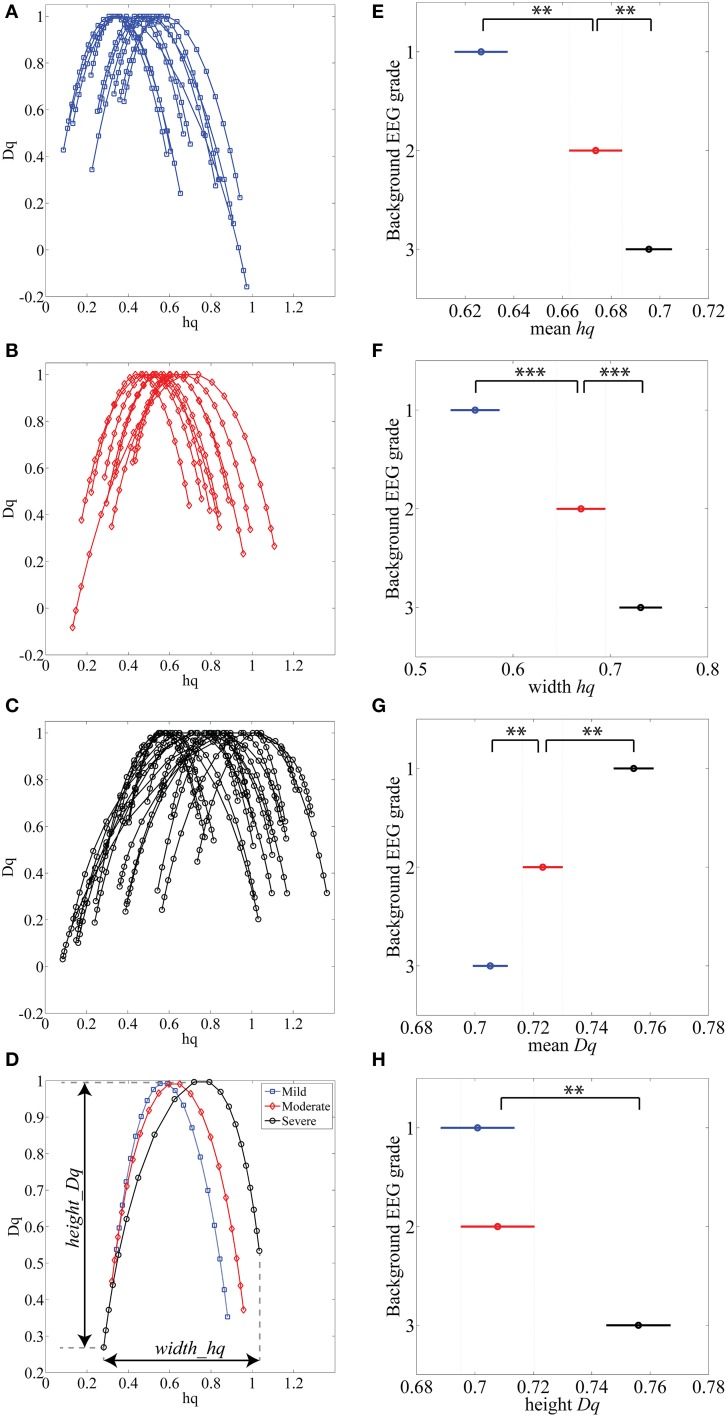
**(A–C)** MF-DFA spectra lines are randomly selected and displayed for various background EEG degrees of abnormality: A-mild (blue), B-moderate (red) and C-severe (black). **(D)** Average of all 1088 multifractal spectra lines is displayed for three background EEG classes. **(E–H)** Results of Kruskal-Wallis mean value group comparisons are displayed for *mean h_q_* (*p* < 0.01), *width h_q_* (*p* < 0.001), *mean D_q_* (*p* < 0.01) and *height D_q_*(not significant). ^**^p < 0.01, ^***^p < 0.001.

Various complex systems and physiological time series may show LRTC although they cannot be characterized with a single parameter (monofractal; for a review, see Ihlen, 2012). For instance, there may be clear monofractal dynamics at distinct, narrow time scales bounded by deflections in the log-log fluctuation plot (so called “crossover points”; Kantelhardt et al., 2002). Then, instead of a single linear approximation, the fluctuation function can be better described as a set of linear approximations, yielding different scaling exponents for different time scales. They are called multifractal time series, where the set of scaling exponents, or parameters, are called *q*-order (generalized) Hurst exponents (Ihlen, 2012). For monofractal time series, the *q*-order Hurst exponents will have equal values, whereas in multifractal time series, the *q*-order Hurst exponents will be significantly diversified. A common reason for multifractality is that the signal may exhibit different LRTC for temporally *local* small and large fluctuations. In contrast, monofractal time series are mainly noise like (e.g., white noise; cf. Ihlen, 2012) and they do not show *local* periods of either extremely small or large fluctuations. Hence, the known non-stationarity of the fluctuations in the neonatal EEG does, indeed, suggest that MF-DFA may offer better handling of the temporal EEG dynamics (e.g., local suppression and burst periods). Mathematically, MF-DFA differs from the DFA only in the calculation of the *q*-order fluctuation functions, *F_q_* (Figures 2B1,B2), which is followed with the extraction of the *q*-order Hurst exponents (Figures 2C1,C2; see also Appendix A). Next, MF-DFA can be plotted as a so called multifractal spectrum, which can be quantified with mathematically robust metrics. The conventional MF-DFA metrics included *mean h_q_*, *width h_q_*, *mean D_q_*, and *height D_q_* (see Figure 3D for their graphical presentation). The *width*_*h_q_* is calculated as the difference between maximal and minimal *h_q_* values, whereas *height*_*D_q_*is calculated as the difference between maximal and minimal *D_q_*values (as suggested in Zorick and Mandelkern (2013). Mean *h_q_*and mean *D_q_* are calculated by averaging all data points across spectra and across 12 channels. The statistical results are presented in Figures 3E–H.

In addition, the difference between DFA and MF-DFA can be observed only within the relatively short time scales (e.g., less than 100 s) and within MF-DFA concept the linear fitting from 1 s till 128 s was applied in this work.

### Benchmarking with EEG grades

The ultimate goal in the present study was to establish the applicability of DFA or MF-DFA metrics in quantification of background EEG abnormalities. To this end, we had two benchmarking methods, visual and automated background classification.

Both DFA exponent and four MF-DFA metrics were calculated exactly the same way for each 15 min EEG epochs that also had a known visually scored background EEG grade inherited jointly for all four 15 min epochs within the given hour of EEG.

*The visual background grading* was performed by an experienced clinical neurophysiologist (PJC), blinded to the clinical details of the neonates. A three level EEG grading was adapted from Murray et al. (2009) as follows: (a) normal/mild (IBIs < 5 s; poorly organized sleep-wake cycle; recovered, continuous background EEG), (b) moderate (5 s ≤ IBIs < 10 s), and (c) severe (10 s ≤ IBI s< 60 s; dominated by prolonged and marked suppressions).

*The automated background grading* was taken from the automatically computed inter-burst intervals (IBI). This was generated using the automated IBI detection method described in Matic et al. (2012). The main steps of this algorithm are summarized in the Appendix B. Subsequently, we used mean IBI values and compared them with MF-DFA based parameters. All data points were extracted from 15 min cEEG epochs, thereby providing for every neonate 32 data values = 4 (epochs of 2 h) × 2 (h) × 4 (15 min segments in 1h cEEG).

#### Statistical analysis

Group comparisons between background grades were performed with the Kruskal-Wallis test, and we applied Bonferroni correction for multiple comparisons for the *post-hoc* tests. There were always three comparisons, hence the corrected significance level were *p* = 0.05/3 = 0.01 and *p* = 0.005/3 = 0.001. Correlations between MF-DFA metrics and IBI values were computed using Spearman correlation coefficient, and here we used more stringent significance level set to 0.01. Binomial statistics were used to evaluate whether significant findings in individual subjects can be considered statistically significant on the group level (see also Vanhatalo et al., 2004; Palva et al., 2005).

## Results

### DFA

#### Visual observations

DFA fluctuation function plots (an example shown in Figure 1C) were computed and examined first visually from hundreds of EEG segments. As a general observation, the ranges of window lengths with possible linear relationships were variable and limited. Our systematic qualitative comparison of window lengths ranging from 5–60 to 10–300 s (data not shown) suggested that linear fit should preferably be attempted at time scales of up to about 60 s. Then, we also reasoned that the lowest feasible limit should be around 10 s to avoid measuring trivial autocorrelations in the amplitude fluctuations (for further reasoning regarding the choice of minimum length, please see Hardstone et al., 2012). A direct comparison of hundreds of DFA fluctuation plots from EEG epochs with different EEG backgrounds showed that they all follow the same overall course, however, they also appear to differ in the segment corresponding to the shorter time windows (Figure 1E).

#### Testing the feasibility of DFA

In above, we had defined the epoch length to 900 s, which was a partly theoretical, partly practical choice. It is known that the DFA fluctuation plots provide more stable estimates of variance when longer epochs are analyzed. The practical reasoning was that the epoch length could be chosen as a multiple of our manual EEG grade classification that was set to 1 h. Obviously, a 900 s long, fixed epoch lengths cannot follow the more rapid dynamic changes known to take place in brain state of these babies (see also Berthouze and Farmer, 2012). Hence, other epoch lengths may be worth trying in future neonatal applications.

The actual feasibility of conventional DFA was tested by examining whether linear fit can adequately characterize the fluctuation plot within the tested range of time scales.

First, our analysis of 1088 epochs using the “maximum likelihood”-based technique suggested that only minority of them could be modeled with a linear fit. This finding was consistent even when we systematically varied frequency band (1–3, 1–5, and 3–8 Hz) or the range of fitting scale (5–60, 10–60, 10–100 and 10–300 s). The best set of parameters, which we fixed and consistently used in the study, was 3–8 Hz frequency band and 10–60 s window fitting size, and even then, only 200 out of 1088 15 min cEEG epochs (18%) were successfully modeled as linear. We also found that continuous background EEG (grade 1) is most likely to fit linear model (data not shown). The detailed results are presented in the Supplemental Figure S1.

Second, we also studied the more commonly applied measure, the linear fit *R*^2^ using the previously adopted threshold of *R*^2^ = 0.97. We found that about two thirds (68%; 731 out of 1088) of EEG epochs passed this test. For detailed comparison of *R*^2^ values and maximum likelihood—based models, please see Supplemental Figure S4.

#### Benchmarking with clinical EEG grades

For benchmarking with clinical EEG grades, we chose only those EEG epochs that had passed our linear fitting test (*R*^2^ > 0.97), and we computed their DFA exponents (α). Group-wise comparison of α shows a wide variation within all EEG grades, while the mean α increases toward more severe EEG background. Notably, the normal/mild EEG grade (continuous EEG activity) was close to α = 0.5, Figure 1D, (an “uncorrelated” process; cf. Hardstone et al., 2012), while the most severe EEG background approached 1.0. Statistical group comparison of α shows that group 3 is significantly different from the other two groups (*p* < 0.01 after *post-hoc* correction; Kruskal-Wallis test).

#### Crossover points

Finally, we visually inspected a large number of DFA plots to observe possible systematic differences between EEG grades. Figure 1E shows an example where the plots have different overall forms: Those computed from the severe EEG grade epochs (black) are at the top, and the plots from the mild EEG grades (blue) are on the bottom of the group. It was also apparent, that the range of linear-appearing trends varied between EEG grades, and that they were often clearly multiphasic: short time scales with steep rise, middle window lengths with flatter rise, and again steep rise at the longest time scales. An example of an EEG epoch with very clear “crossover points,” i.e., change of slope in the fluctuation function (Hardstone et al., 2012) is shown in Figure 1F. Finding such crossover points, suggests that the signal might exhibit multifractal behavior (Kantelhardt et al., 2002; Ihlen, 2012; however, see Hu et al., 2001). Describing such signal with one exponent, as in the conventional monofractal DFA, will overlook key dynamic properties, and the results will be particularly sensitive to chance factors in the conventional DFA analysis.

In our dataset, the crossover point in the burst-suppression background (red and black DFA fluctuation functions in Figure 1E) was usually encountered within 30–60 s time scale [1.5–2 in log_10_(time) s]. Continuous EEG background (blue DFA fluctuation functions) were successfully modeled as linear for longer window sizes [up to 100 s or 2 in log_10_(time) s], and the crossover points mainly appeared in the interval of 100–300 s [2–2.5 in log_10_(time) s]. In comparing different EEG grades, the different crossover points hence preclude use of single, monofractal DFA exponent, and supports exploration of MF-DFA metrics.

### Multifractal DFA (MF-DFA)

We next studied the feasibility of MF-DFA in neonatal EEG by applying a previously published method (Kantelhardt et al., 2002; Ihlen, 2012). The special clinical attraction in MF-DFA comes from its clear metrics that have been recently shown to aid discrimination of brain states (Zorick and Mandelkern, 2013) and pathologies (Zheng et al., 2005).

#### Feasibility of MF-DFA

The first step here was to show that neonatal EEG signal is characterized by multiple rather than single exponents. The presence of multifractal behavior in at least some neonatal EEG data becomes apparent when comparing the temporal behavior of two common neonatal EEG patterns in asphyxiated infants, the normal continuous EEG signal and the severely abnormal EEG with marked intermittency called burst-suppression (see Figures 2A1,A2). For those signals, we calculated the *q-order* fluctuation functions *F_q_* (Figures 2B1,B2) with *q* values from −5 till 5, equidistantly sampled with a 0.5 unit-step as suggested by Ihlen (2012), notably, different range could be examined as well. The *q* factor is able to detect mixed occurrence of large fluctuations with periods of negligible fluctuations, which in our case characterizes the burst-suppression background. In contrast, the continuous EEG consists of neither very large nor very small fluctuations (Figure 2A1).

Comparison of *q*-order fluctuation functions (*F_q_*) taken from these two different EEG patterns shows their fundamental difference. In the normal, continuous EEG activity, the slopes of the fluctuation functions appear comparable to each other. Hence, they could also be reasonably described with only one DFA fluctuation function (Figure 2B1; *q* = 2), and be considered as monofractal process. In contrast to this, EEG periods with burst-suppression are much more variable, and different time epochs exhibit a notably wide spread of *F_q_* (Figure 2B2), suggestive of an underlying multifractal process (see also Ihlen, 2012). Taken together, these observations suggest that measuring LRTC in neonatal EEG datasets like ours should require quantification of multifractal dynamics.

Subsequently, we calculated *q*-order Hurst exponents, *H_q_* (Ihlen, 2012), which returns linear approximation of the *F_q_* fluctuation functions (Figures 2C1,C2). This again discloses a clear difference between background EEG activity grades. In the continuous EEG, the relation between *H_q_* and *q* factor shows a monotonic decrease (characteristic of a monofractal process; Ihlen, 2012), whereas the *H_q_* plot of burst-suppression EEG (Figure 2C2) shows a relatively sharp and nonlinear decline (characteristic of a multifractal process). These *q*-order Hurst exponent functions can be readily transformed into a multifractal spectrum (Figures 2D1,D2), which outlines the relation of local temporal changes of the *H_q_* and the distribution of its probability density function (see Ihlen, 2012). Multifractal spectrum represents two measurable dimensions, the *D_q_* (*q*-order singularity/fractal dimension) and the *h_q_* (*q*-order singularity exponent). In the visual assessment of multifractal spectra, differences can be noticed in a horizontal and vertical positioning (*h_q_*, *D_q_* values), a width (*width_h_q_*), as well as in the general shape of the multifractal spectra reflecting temporal variations of local Hurst exponents.

The MF-DFA spectra were then computed for each (*n* = 1088) of the 15 min long EEG epochs. Example of these spectra are plotted from each EEG background grade (Figures 3A–C). Visual inspection of the spectra shows that worsening of EEG background (from mild to severe) is associated with a shift to the right (higher *h_q_* value). Grand averages of the spectra are shown in Figure 3D (1088 epochs from 12 channels: total 13056 spectra).

Other differences among EEGs were also observed visually within the tails of the spectra: the left tails, corresponding to positive *q* values, are longer in the EEG with local large fluctuations, i.e., the severe EEG grade with IBIs > 10 s (Figure 3C). Moderate and milder EEG background traces do not have such pronounced structural differences and the corresponding tails are respectively shorter (Figures 3A,B). On the other hand, the right tails, reflect the amplitude fluctuations within local periods of low activity (e.g., suppressed EEG periods).

#### Benchmarking with clinical EEG grades

Comparison of MF-DFA metrics between EEG grades showed highly significant differences between the three EEG grades. The mean height of spectra, *mean h_q_*, was significantly (*p* < 0.01; Kruskall-Wallis test) increased with EEG severity (mild 0.63 ± 0.10; moderate 0.67 ± 0.09; severe 0.70 ± 0.09). The width of the spectra, *width h_q_*, was also significantly (*p* < 0.001) increased with increasing severity of EEG background (mild 0.56 ± 0.13; moderate 0.67 ± 0.16; severe 0.73 ± 0.19). At the same time, the *mean D_q_* decreased significantly (*p* < 0.01) with increasing severity of EEG background (mild 0.75 ± 0.05; moderate 0.72 ± 0.05; severe 0.70 ± 0.06). Height of the MF-DFA spectra, *height_D_q_*, was significantly (*p* < 0.01) different between moderate and severe EEG grades (moderate 0.70 ± 0.09; severe 0.76 ± 0.11). These findings together suggest that MF-DFA is superior to the conventional DFA in distinguishing between neonatal EEG grades.

A further inspection of the shapes of MF-DFA spectra shows additional qualitative spectral features that are different between background grades. Namely, the mild/normal EEG grade does typically correspond to asymmetric spectra with a longer right side tail, whereas the spectra of severe EEG grade are more asymmetric with longer left side tail. This asymmetry is not measured by the currently used, established MF-DFA metrics. It may, however, readily explain the lesser differences between groups seen in *mean_D_q_* and *height_D_q_*, which are insensitive to asymmetry. To examine this further in a quantitative manner, we also computed the metrics from the right side tail only (see below).

#### Benchmarking and application into intraindividual context

While the group level findings above are by themselves strong, clinical applicability of any novel study paradigm requires performance at individual level as well (see discussion in Ahtola et al., 2014). In order to preliminarily probe this issue, we studied those 34 neonates in our dataset that had four EEG epochs of 2 h duration randomly selected at time points with at least 4 h intervals. We then computed MF-DFA metrics from 15 min segments within each 2 h epochs (total 32 epochs in each infant). The long intervals between epochs yielded us datasets where EEG grade, or at least the mean IBI, was changing due to variation in the clinical state of the patient. As a benchmark to MF-DFA metrics, we calculated the mean IBI for each 15 min epoch using our previously described automated detector (Matic et al., 2012). Nine patients were excluded because their range of IBIs was too narrow to reasonably compute correlation coefficients with another parameter, i.e., IBI range was only within 3 to 6 s interval. This left 25 babies for our analysis. Finally, non-parametric, ranking correlation (Spearman) was computed between mean IBI levels and MF-DFA spectra metrics in each patient.

#### Measures of the full MF-DFA spectra

**Figure 4:**
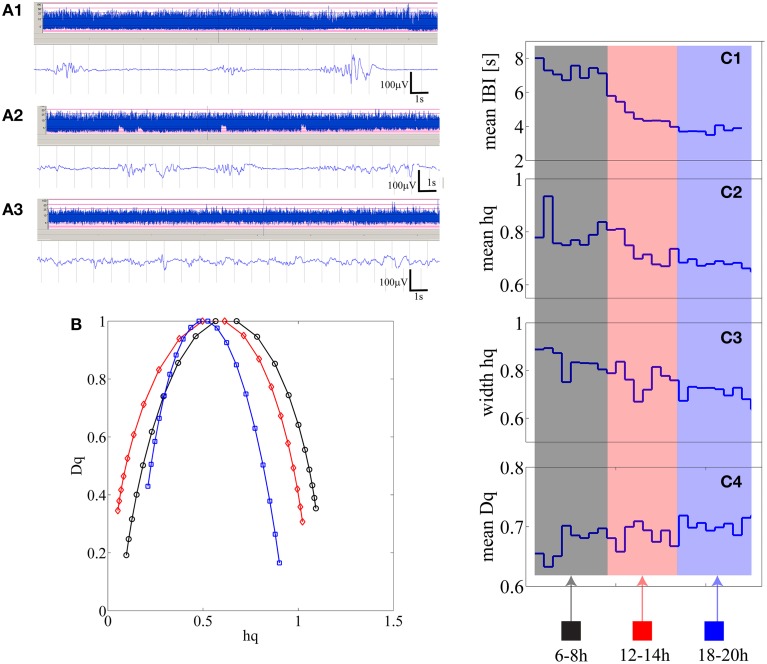
**(A1–A3)** Three examples of burst-suppression, tracé discontinue, and continuous EEG periods are shown as aEEG trends and EEG epoch in an infant who is showing recovery from initial severe hypoxic insult. **(B)** Three multifractal spectra lines are shown for the corresponding three states quantifying 15 min of burst-suppression (black-recorded at 6 h post-partum), tracé discontinu (red-recorded at 12 h post-partum) and continuous EEG period (blue-recorded at 18 h post-natal age). (**C1–C4**) from 15 min segments mean IBI, *mean h_q_*, *width h_q_* and *mean D_q_* values are represented as one data point [24 in total; 2 h × 4 (15 min epochs in 1 h) × 3 epochs].

Individual results are shown in Supplementary Table 1, Supplemental Data. We observed that in 10 out of 25 babies there was a significant (*p* < 0.01) correlation at the individual level, which finding is statistically very significant at the group level (*p* < 1e–10; Binomial statistics). As shown in Figure 4, for an example infant (*n* = 21), a visually apparent temporal relationship was observed between decreasing IBI trend and changes in at least three of the MF-DFA parameters (*mean_hq*, *width_hq*, and *mean_Dq*).

#### Measures of the right side tail of MF-DFA spectra

Closer inspection showed that the signs of correlation coefficients (*ρ*) were inconsistent even for infants with statistically significant correlations (Supplementary Table 1, Supplemental Data). To search for reasons we inspected further the shapes of MF-DFA spectra, and observed their asymmetry, especially in the severe EEG grades. For instance, visual comparison of individual MF-DFA spectra shown in Figure 4B from different time epochs during recovery shows that the shape of spectra changes over time, most notably as a shift from longer left tail to longer right tail as well as the shift of the right tail to the left when going from severe to normal EEG background, respectively. Since the current MF-DFA metrics are insensitive to asymmetry of the multifractal spectra, it is possible that change in symmetry with EEG grades would confound the correlations. Hence, we investigated the parameterization of the right tail of the MF-DFA spectra, and we found these metrics to exhibit much stronger relationship to the mean IBI values. In particular, for 13 out of 25 patients, we found that at least 3 metrics namely *width hq* (ρ = −0.70 ± 0.15), *mean Dq* (ρ = 0.68 ± 0.13) and *height Dq* (ρ = −0.67 ± 0.11), were significantly correlated with the mean IBI values, showing consistent range of correlation coefficient values (ρ) as detailed at subject level in Supplementary Table 2, Supplemental Data.

**Figure 5:**
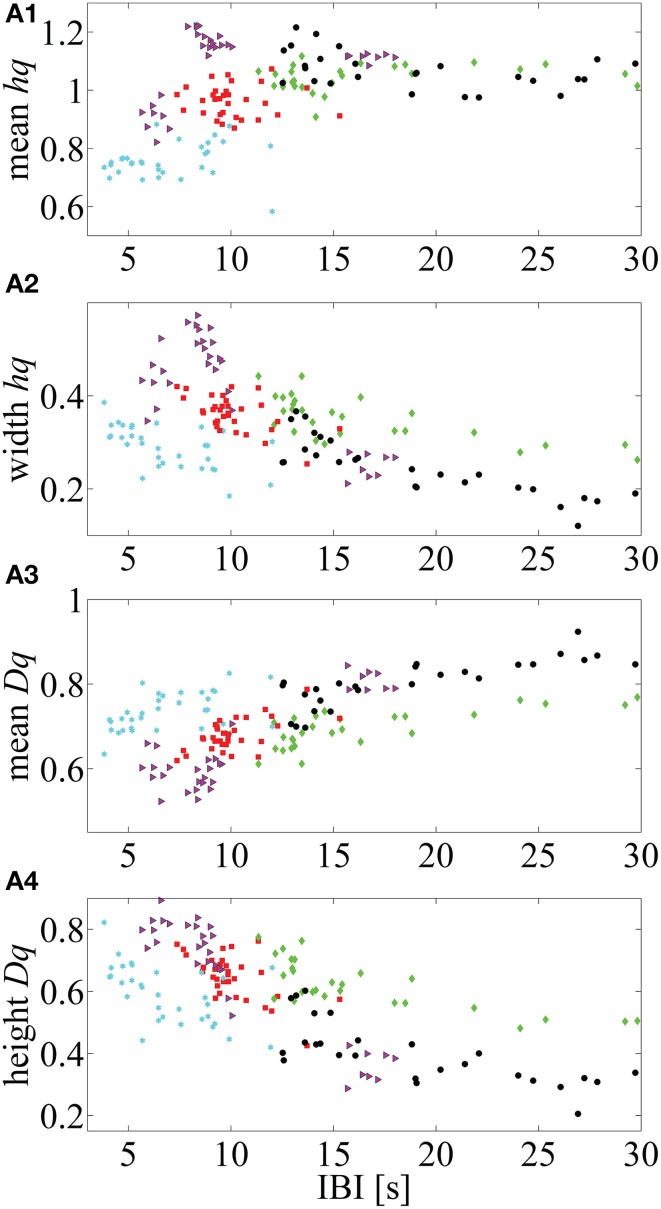
**Five neonates in which mean IBI values are highly correlated with the MF-DFA metrics (calculated from the right part of the multifractal spectra)**. **(A1–A4)** respectively, represent the correlation between *mean h_q_*, *width h_q_*, *mean D_q_* and *height D_q_* values with the mean IBIs. This approach for calculation of MF-DFA based metrics avoids insensitivity to the asymmetry which affects the originally proposed parameters calculated for the complete spectra (see Zorick and Mandelkern, 2013).

Additionally, we illustrate how the MF-DFA parameters (extracted from the right tail of the spectra) are correlated with IBI values for five infants that showed higher Spearman correlation *p*-values (selected from the Supplementary Table 2, Supplemental Data) (Figure 5).

## Discussion

Our study shows that changes in long range temporal dynamics in the early neonatal EEG may be characterized with the DFA paradigm, however the conventional DFA is compromised and the recently introduced multifractal DFA can better disclose neonatal EEG dynamics in a way that correlates with the current clinical EEG grading systems. Our clinical benchmarking of DFA feasibility extends prior literature to include full-term neonatal EEG as a potential application of DFA/MF-DFA paradigms. The present findings corroborate earlier reports that DFA or MF-DFA metrics can quantify brain dynamics associated with prematurity (Hartley et al., 2012) and neurological conditions (Linkenkaer-Hansen et al., 2001; Parish et al., 2004; Monto et al., 2007). We also suggest a novel MF-DFA metric for EEG analysis based on observing asymmetric change in MF-DFA spectra between clinically relevant brain states.

The physiological underpinnings of multifractal behavior in the neonatal brain remain elusive, and our dataset is suboptimal for a tour de force exploration of the phenomenon of multifractality in the neonatal brain activity. However, our observations provide some tentative ideas to how MF-DFA may be suitable in such data: The well-known hallmark of both the early preterm EEG and the severely abnormal EEG of a term newborn is a salient intermittency (Vanhatalo and Kaila, 2006; André et al., 2010): seemingly erratic ruptures of transient cortical activity alternate with periods of relative quiescence. Considering the structural immaturity of underlying brain networks (Kostović and Judaš, 2010) and/or the pathological mechanisms related to resource constraints (Roberts et al., 2014), it is conceivable that they would readily compromise the extreme long range temporal correlations previously shown in the adult brain (Linkenkaer-Hansen et al., 2001). There is ample evidence from the neonatal EEG studies that cortical activity may continue even after extensive structural lesions that lead to physical disconnection of cortical areas. The multifractality could hence reflect a spatial and/or temporal fragmentation of large scale brain activity, resulting in multiple partly independent mechanisms in brain activity. Nevertheless, more rigorous confirmation with more extensive datasets and stringent mathematical testing of alternative models will be necessary to confirm fundamental behaviors of this kind (for instance, see Clauset et al., 2009; Roberts et al., 2014).

In addition to showing the significant discrimination of background grades with the established MF-DFA metrics, our closer visual inspection of MF-DFA spectra suggested further differences. The highest difference between grades was achieved for the relatively small window size values [<30 s or 1.5 in log_10_(time)s] (Figure 2B2), which is most sensitive to temporally *local* variations in signal fluctuations. We also observed that the different background grades yield saliently different shapes, especially the asymmetry, in their corresponding MF-DFA spectra. This is not quantified by the established MF-DFA metrics (cf. Zorick and Mandelkern, 2013), hence we proposed quantification of the right side tail of the spectra for an improved sensitivity. These measures were, indeed, significantly correlating with IBIs in a large proportion of infants. It is possible that the prominent intermittency of the burst suppression signal has an important effect on the MF-DFA metrics. Conceivably, burst suppression EEG consists of two overlaid, but at least partly independent physiological processes: intermittent bursting behavior and the varying levels of spontaneous activity between the bursts, both with unique scaling properties (Roberts et al., 2014). A similar situation is also found during the early intermittent EEG activity of preterm babies where two mechanistically different brain activities are superimposed on each other (Vanhatalo and Kaila, 2006), giving rise to unique long range temporal correlations in the inter-event time series (Hartley et al., 2012). The physiological meaning of asymmetry remains to be established in simulations and experimental studies, however our data suggests that it relates to continuity and perhaps the relative shapes of bursts that interrupt the interburst silence.

In search of clinical relevance, we benchmarked the MF-DFA metrics with both visual and automated background EEG grading, and show that all our four metrics (*mean h_q_, width h_q_, mean D_q_, height D_q_*) do significantly co-vary with the visually scored EEG background grades. Three (*mean h_q_, width h_q_, mean D_q_*) of our metrics were significantly different between all EEG groups. In addition, the application within patients showed that MF-DFA metrics might even reflect temporal change of background EEG states at individual level as suggested by the significant correlations with IBI levels in 10 out of 25 infants. These correlations suggest that MF-DFA metrics and IBI would at least partly reflect similar pathological brain dynamics, or at least the amplitude fluctuations of burst suppression are reflected in the MF-DFA spectra. However, despite the general correspondence with IBI levels and its evolution, the match between IBI and MF-DFA metrics is neither fully consistent nor complete. Such partial mismatch are consistent with the idea that MF-DFA metrics reflect some as yet latent EEG signal dynamics.

Beyond the physiological interest, our present findings have potential implications in future construction of computational classifiers for neonatal EEG. A number of studies have reported development of various automated EEG background classifiers. Some of them are designed to directly replicate the visual criteria. Using this approach, the recent study by Korotchikova et al., 2011, combined a larger set of quantified EEG features and found them indiscriminative with respect to background EEG classes, with special challenges in distinguishing mild vs. moderate grades. A more recent attempt along this line was able to discriminate normal vs. abnormal EEG backgrounds as a dichotomous choice (Stevenson et al., 2013), which does not yet support its use in EEG monitoring or for more refined needs. Another approach has been to optimize a learning algorithm with a smaller dataset to detect extreme classes of EEG background, such as severe burst-suppression patterns (Löfhede et al., 2010), which is further used to output IBI detections. The design of such classifier will ideally allow accurate quantitation of IBIs (Flisberg et al., 2011), however, it will not allow constructing methods that can monitor evolution of EEG activity over background grade boundaries, which obviously happens among a majority of clinical patients during their brain monitoring.

Prior extensive engineering works have mostly focused on identifying discrete EEG states (e.g., burst suppression), or on discrimination of normal from abnormal backgrounds as benchmarked with visual criteria. One of the shortcomings in benchmarking with visual EEG grading is its temporal coarseness. Visual grades are commonly assigned hourly for slow changes such as sleep-wake cycling (Murray et al., 2009; Matic et al., 2014; Stevenson et al., 2014). Other EEG grading systems have been developed to include even longer term evolution, up to over 24 h, to characterize recovery from hypoxic insults (Cherian et al., 2011), which as a principle reminds of the conventional approach used in the aEEG assessment (Hellström-Westas and Rosén, 2006). We are not aware of objective computational, graded measures of background EEG activity although the clinical experience shows that the key part of brain monitoring is the follow-up of patients through different stages over hours. Our findings suggest that measures based on MF-DFA metrics may offer an advantage here: for instance, these metrics may distinguish moderately abnormal EEG from the severe and normal backgrounds, which is clinically very important. Brain states change more rapidly in the real life, hence we wanted to compare MF-DFA-based metrics also with the automated algorithm that detects IBI levels in 15 min epochs. This showed that MF-DFA metrics do co-vary with IBI, and they do evolve over time. A systematic characterization and benchmarking with existing trend paradigms, especially aEEG (Hellström-Westas and Rosén, 2006) will help to identify the clinical added value of MF-DFA measures.

We consider our study as exploratory, providing only proof of concept. Further prospective and larger studies are needed to determine the clinical robustness and added value obtained from the proposed method, as well as to validate its use in the prospective clinical context. The first practical consideration is the age range of infants. Our dataset was limited to full-term neonates, but we do not see any *a priori* physiological or technical reason why the same temporal dynamics could not be clinically meaningful in the preterm babies as well. The second practical consideration is the unknown relative sensitivity of our metrics to artifacts that are unavoidably present in real life recordings from intensive care units. This can be assessed formally by simulated addition of real EEG artifacts (cf. Matic et al., 2009; De Vos et al., 2011; Räsänen et al., 2013). The future artifact avoidance strategies may include rejection by amplitude criteria (cf. Palmu et al., 2013), although missing data segments may also introduce confounders. The third practical consideration is the added clinical value relative to existing measures, especially the aEEG paradigm. It is conceivable that our metrics provide a theoretically and functionally complementary view on neonatal brain dynamics, yet the actual added bedside value needs to be determined in future clinical studies. There are also technical considerations for future studies. First, the known alternative ways in the MF-DFA analysis, such as wavelet transform modus maxima (Muzy et al., 1993), might be useful to further improve the robustness and consistency (Zorick and Mandelkern, 2013). Second, our study demonstrate as yet uncharacterized changes in the spectral shape, which suggests that further theoretical and practical studies are needed to define spectral measures that optimally reflect changes in the neurophysiological and/or clinical state of the brain.

### Conflict of interest statement

The authors declare that the research was conducted in the absence of any commercial or financial relationships that could be construed as a potential conflict of interest.

## Supplementary material

Figure B1 **Steps of the automated IBI detection method are illustrated**. First, EEG signal is segmented using an adaptive segmentation algorithm **(A1)** Next, the segments are classified into the low amplitude class (approximately <10 μV) and depicted in blue. **(A2)** The temporal profile signal counts the prevalence of low amplitude segments and it is used to detect IBI patterns **(A3)**. The time that the temporal profile signal exceeds the threshold determines the IBI duration and the IBI is detected.
