# Supplementary material for: Objective differentiation of neonatal EEG background grades using detrended fluctuation analysis
Source: Front Hum Neurosci. 2015 Apr 23;9:189. doi: 10.3389/fnhum.2015.00189 (PMC4407610; doi:10.3389/fnhum.2015.00189)
Supplement: Figure B1 — Steps of the automated IBI detection method are illustrated. First, EEG signal is segmented using an adaptive segmentation algorithm (A1) Next, the segments are classified into the low amplitude class (approximately <10 μV) and depicted in blue. (A2) The temporal profile signal counts the prevalence of low amplitude segments and it is used to detect IBI patterns (A3). The time that the temporal profile signal exceeds the threshold determines the IBI duration and the IBI is detected. [file DataSheet1.DOCX]

***Supplementary Material***

**Objective differentiation of neonatal EEG background grades using detrended fluctuation analysis**

**Vladimir Matic^1,2^, Perumpillichira J Cherian^3^, Ninah Koolen^1,2^, Amir H Ansari^1,2^, Gunnar Naulaers^4^, Paul Govaert^5^, Sabine Van Huffel^1,2^, Maarten De Vos^6^, Sampsa Vanhatalo^7,8^**

^1^KU Leuven, Department of Electrical Engineering (ESAT), STADIUS Centre for Dynamical Systems, Signal Processing and Data Analytics,

Leuven, Belgium

^2^iMinds Medical IT Department, Leuven, Belgium

^3^Section of Clinical Neurophysiology, Department of Neurology, Erasmus MC, University Medical Center, Rotterdam, The Netherlands

^4^Neonatal Intensive Care Unit, University Hospital Gasthuisberg, Leuven, Belgium

^5^Section of Neonatology, Department of Pediatrics, Erasmus MC-Sophia Children’s Hospital, University Medical Center, Rotterdam, The Netherlands

^6^Institute of Biomedical Engineering, Department of Engineering, University of Oxford, Oxford, UK

^7^Department of Clinical Neurophysiology, Children’s Hospital, Helsinki University Central Hospital, University of Helsinki, Helsinki, Finland

^8^Department of Neurological Sciences, University of Helsinki, Helsinki, Finland

*** Correspondence:** Vladimir Matic, Division STADIUS-BIOMED, Department of Electrical Engineering, KU Leuven, Kasteelpark Arenberg 10 - bus 2446, 3000 Leuven, Belgium.

[vladimir.matic@esat.kuleuven.be](mailto:vladimir.matic@esat.kuleuven.be) and [maticvl@gmail.com](mailto:maticvl@gmail.com)

1. **Supplementary Data**

**Supplementary Figure 1.**

| **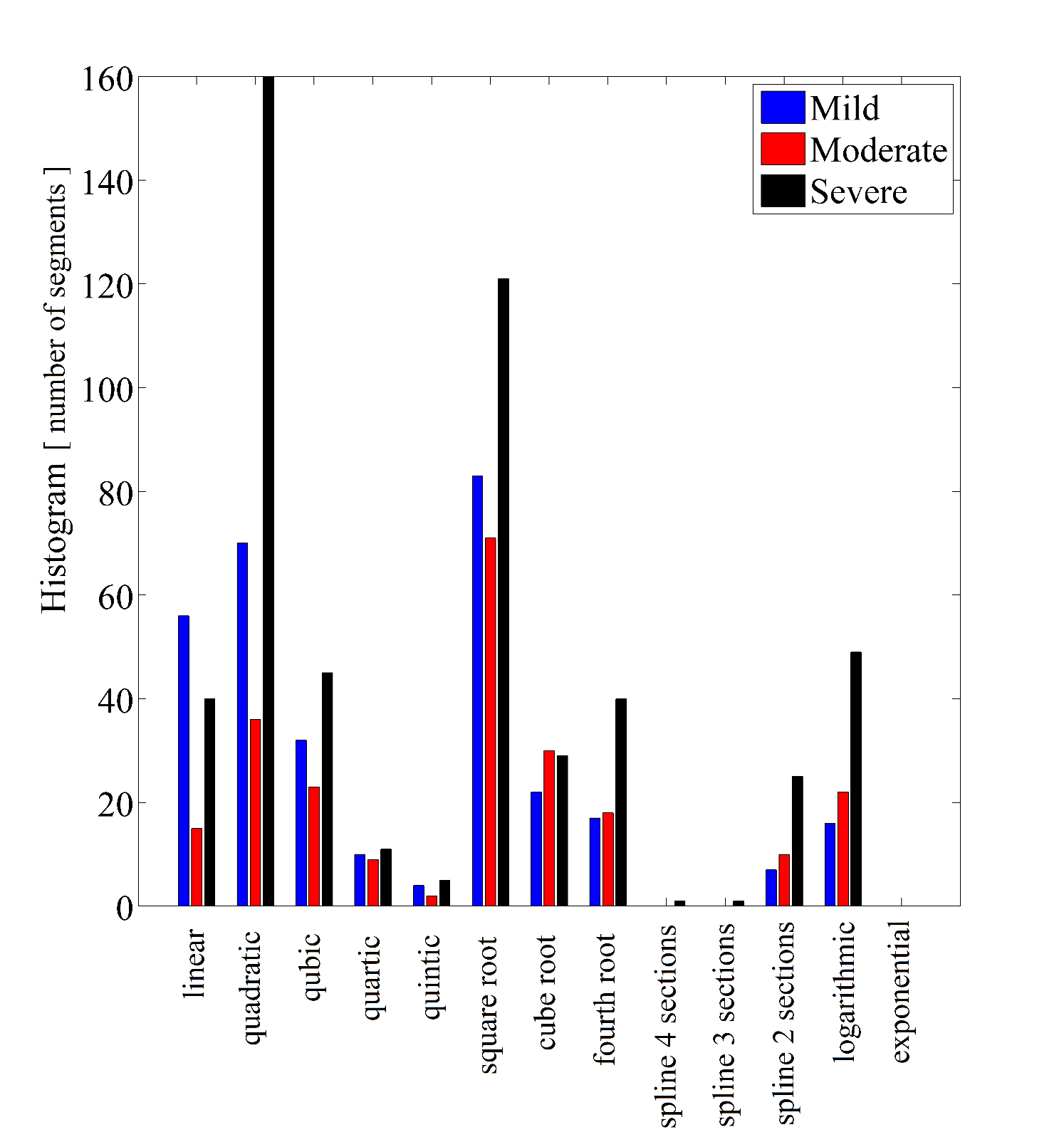** |
| --- |
| **Supplemental Figure S1.** In order to test the feasibility of the DFA onto the neonatal EEG we have applied ‘maximum-likelihood’-based technique. This method examines the DFA fluctuation function fitting within 10-60s time window. Histogram values show the number of EEG segments that were best fitted with one out of 13 optimal models. In addition, we show the results how the EEG segments are fitted with respect to the background EEG grades (mild, moderate, and severe). |

**Supplementary Figure 2.**

| **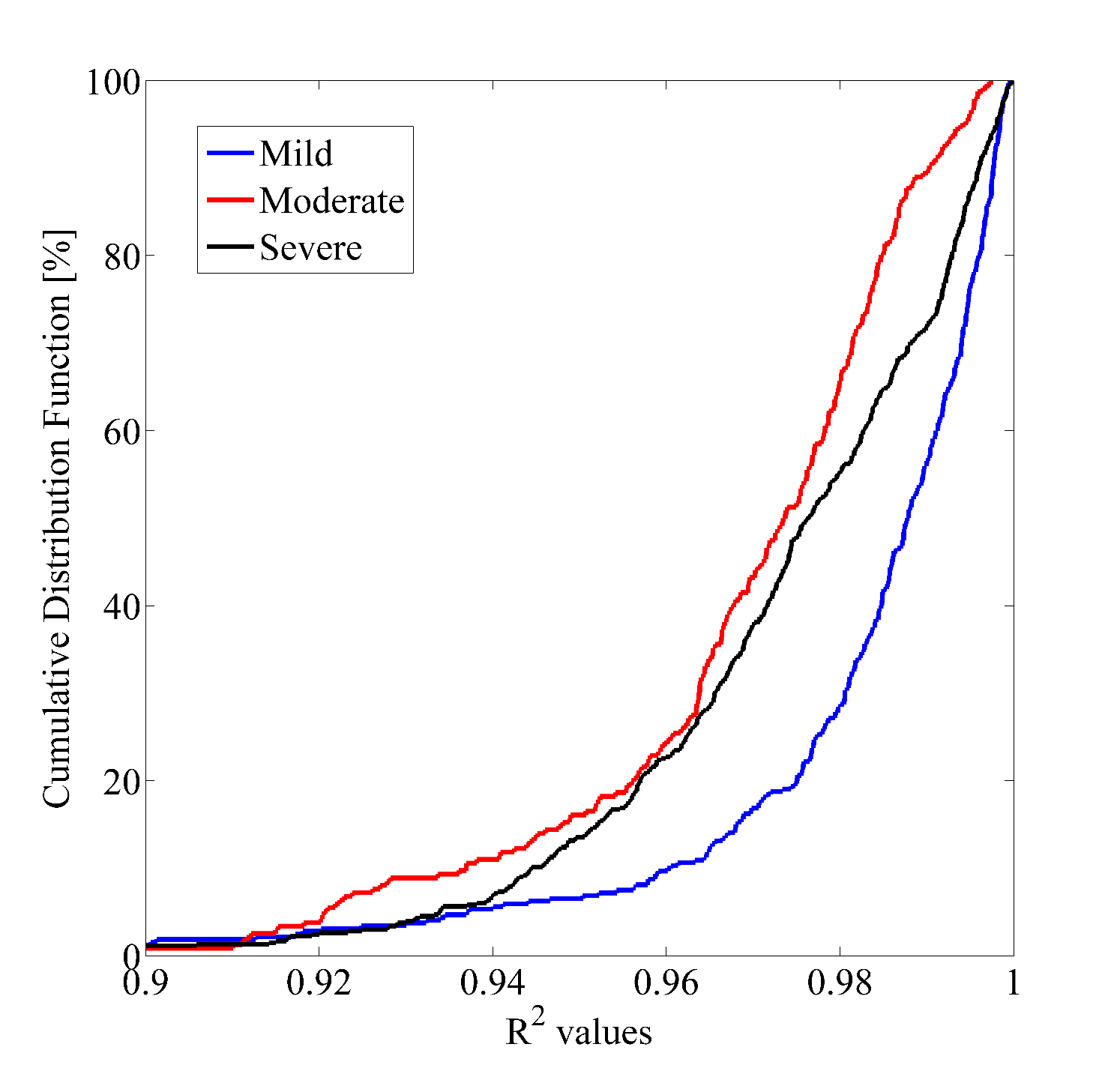** |
| --- |
| **Supplemental Figure S2.** A common measure to examine the linear approximation fit is the linear fit *R*^2^ value. Depending on the three background EEG grades we show the cumulative distribution function of the *R*^2^ values. Mild/continuous background EEG has the highest capacity to be fitted as linear. |

| **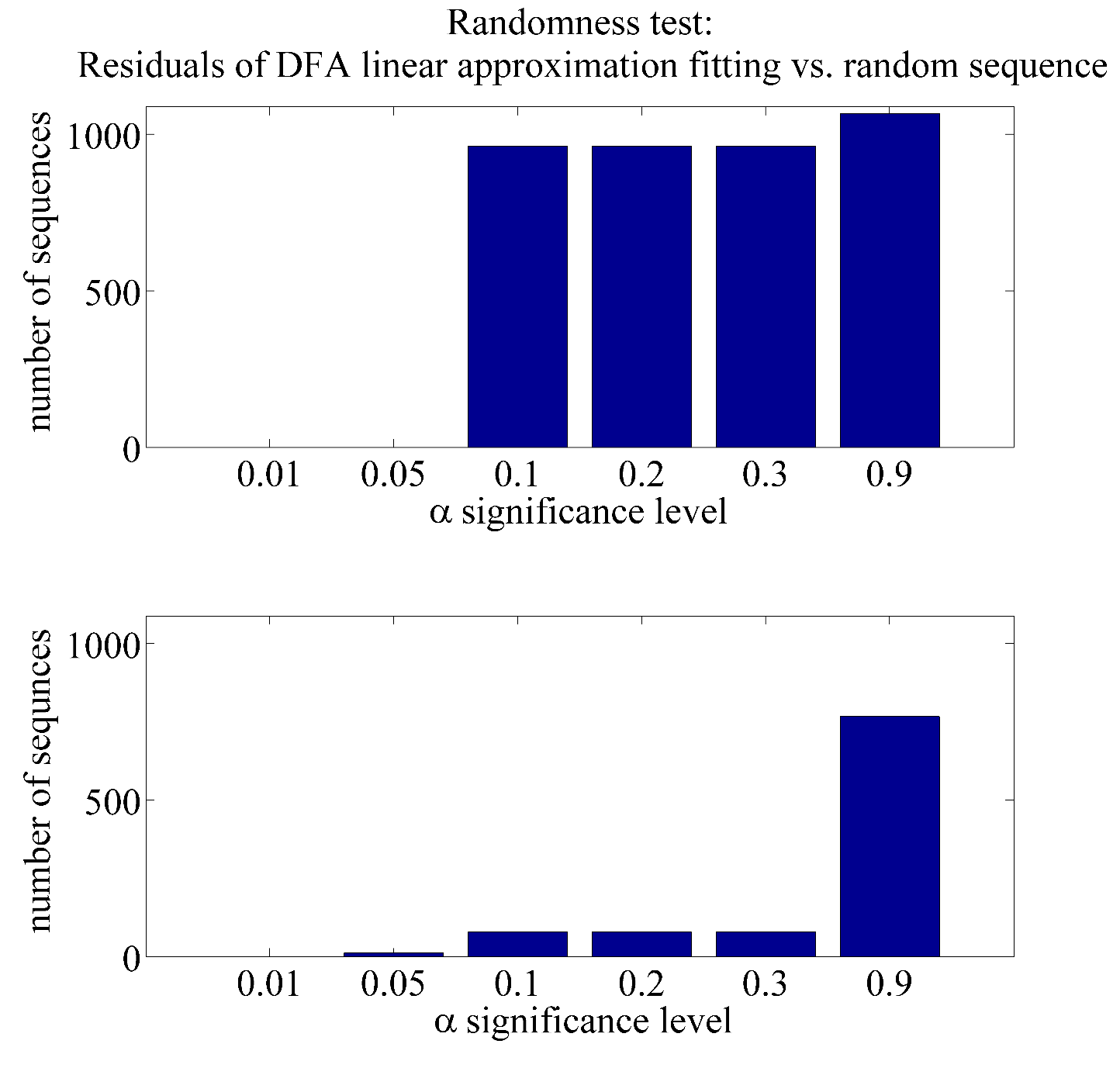** |
| --- |
| **Supplemental Figure S3.** Residuals of the linear approximation fitting have been tested (11 data points) to examine whether they pass the randomness test (upper plot). In addition, the 11 data points of randomly generated sequence are tested using the same method (bottom plot). This has been achieved with the MATLAB function *'runstest'*. The number of segments for alpha values (0.1-0.3) implies that for a large number of EEG samples the residuals do not pass the randomness test. In contrast, for the random data sequence, only a few sequences are not regarded as random. |

| A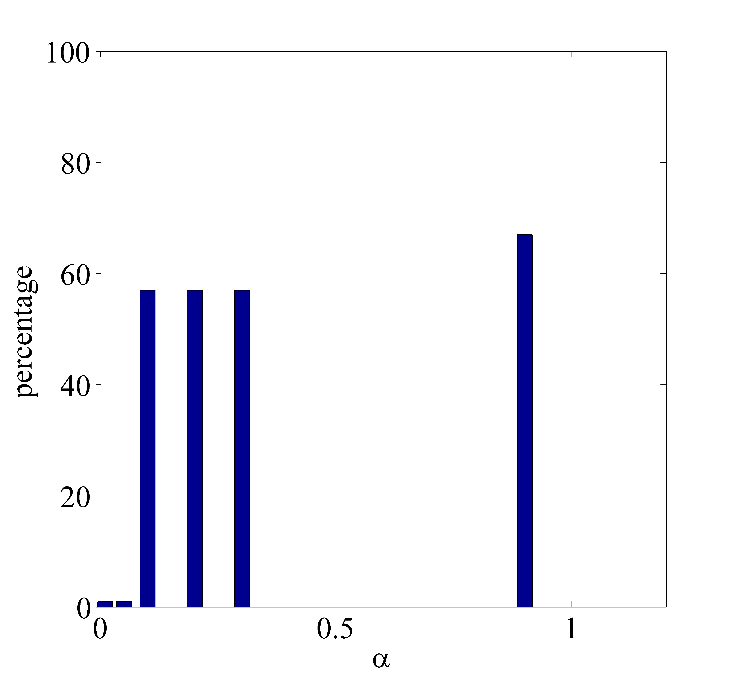 B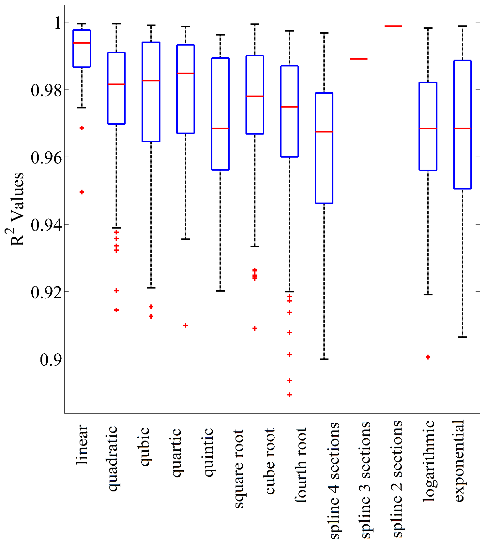 |
| --- |
| **Supplemental Figure S4.** A) Percentages of the epochs having (R^2^>0.97) that did not pass the randomness test. B) Comparison of R^2^ values with the model selected by maximum likelihood test. Expectedly, the linear model had significantly higher R^2^ values than the other models (p<0.001, ANOVA; pairwise posthoc comparison by Bonferroni test) |

**Supplementary Table 1.**

Correlation between IBI and MF-DFA metrics computed from the **whole MD-DFA spectra**. The four left side columns present significance levels and the four right side columns show the corresponding Spearman correlation coefficients. Significant correlations (p < 0.01) are highlighted with green, and their corresponding ρ values are highlighted in orange. The correlations in each patient were computed from 32 epochs.

| *p*-value: Spearman correlation *p* values of MF-DFA metrics with mean IBI values. | | | | | *ρ*: Spearman correlation coefficient values of MF-DFA metrics with mean IBI values. | | | | |
| --- | --- | --- | --- | --- | --- | --- | --- | --- | --- |
| Patient  number | mean *hq* | width *hq* | mean *Dq* | height *Dq* | Patient  number | mean *hq* | width *hq* | mean *Dq* | height *Dq* |
| 1 | <0.01 | 0.24 | 0.15 | 0.63 | **1** | 0.52 | 0.21 | -0.26 | -0.09 |
| 2 | 0.67 | 0.19 | 0.39 | 0.90 | **2** | 0.08 | 0.24 | -0.16 | 0.02 |
| 3 | 0.06 | 0.30 | 0.31 | 0.89 | **3** | 0.34 | 0.19 | -0.18 | 0.03 |
| 6 | 0.02 | 0.43 | 0.69 | 0.61 | **6** | 0.40 | -0.15 | 0.07 | -0.09 |
| 7 | <0.01 | 0.52 | 0.58 | 0.26 | **7** | 0.70 | -0.12 | 0.10 | -0.20 |
| 11 | 0.37 | 0.14 | 0.61 | 0.83 | **11** | -0.16 | 0.27 | -0.09 | 0.04 |
| 12 | <0.01 | 0.30 | 0.39 | <0.01 | **12** | -0.47 | 0.19 | -0.16 | 0.66 |
| 14 | 0.81 | 0.06 | 0.66 | 0.07 | **14** | 0.05 | -0.33 | 0.08 | -0.32 |
| 16 | 0.12 | 0.51 | 0.67 | 0.04 | **16** | 0.28 | 0.12 | 0.08 | -0.36 |
| 17 | 0.10 | 0.80 | 0.61 | 0.01 | **17** | -0.29 | 0.05 | -0.09 | 0.44 |
| 18 | <0.01 | <0.01 | <0.01 | <0.01 | **18** | 0.61 | -0.82 | 0.81 | -0.65 |
| 19 | <0.01 | 0.76 | <0.01 | 0.20 | **19** | -0.51 | 0.06 | -0.51 | 0.23 |
| 20 | 0.06 | 0.27 | 0.18 | 0.01 | **20** | -0.33 | -0.20 | 0.24 | -0.45 |
| 21 | <0.01 | <0.01 | 0.01 | 0.75 | **21** | 0.61 | 0.61 | -0.48 | 0.06 |
| 22 | 0.62 | 0.31 | 0.12 | 0.37 | **22** | 0.09 | -0.18 | 0.28 | -0.16 |
| 23 | 0.65 | 0.06 | 0.08 | 0.05 | **23** | 0.08 | 0.33 | -0.31 | 0.35 |
| 24 | 0.25 | <0.01 | <0.01 | <0.01 | **24** | 0.21 | 0.68 | -0.71 | 0.58 |
| 25 | 0.10 | 0.33 | 0.17 | <0.01 | **25** | 0.30 | 0.18 | 0.25 | -0.71 |
| 26 | 0.01 | 0.01 | 0.25 | <0.01 | **26** | -0.47 | -0.46 | 0.21 | 0.50 |
| 27 | <0.01 | 0.81 | 0.95 | 0.65 | **27** | 0.68 | -0.04 | 0.01 | -0.08 |
| 28 | <0.01 | 0.48 | 0.24 | <0.01 | **28** | -0.57 | 0.13 | -0.21 | 0.68 |
| 30 | 0.32 | 0.74 | 0.83 | 0.57 | **30** | -0.18 | -0.06 | 0.04 | 0.10 |
| 31 | 0.22 | <0.01 | 0.09 | 0.05 | **31** | 0.22 | -0.60 | 0.31 | 0.35 |
| 32 | 0.01 | 0.85 | 0.33 | 0.71 | **32** | -0.44 | 0.03 | -0.18 | 0.07 |
| 33 | 0.80 | 0.58 | 0.94 | 0.45 | **33** | -0.05 | 0.10 | -0.01 | -0.14 |

**Supplementary Table 2.**

Correlation between IBI and MF-DFA metrics computed from the **right side of the MD-DFA spectra**. The four left side columns present significance levels and the four right side columns show the corresponding Spearman correlation coefficients. Significant correlations (p < 0.01) are highlighted with green, and their corresponding ρ values are highlighted in orange. The correlations in each patient were computed from 32 epochs. Comparison to Table 1 shows that the sign of correlation is more consistent when measuring the right side of spectra only.

| *p*-value: Spearman correlation *p* values of MF-DFA metrics with mean IBI values. | | | | | *ρ*: Spearman correlation coefficient values of MF-DFA metrics with mean IBI values. | | | | |
| --- | --- | --- | --- | --- | --- | --- | --- | --- | --- |
| Patient  number | mean *hq* | width *hq* | mean *Dq* | height *Dq* | Patient  number | mean *hq* | width *hq* | mean *Dq* | height *Dq* |
| 1 | <0.01 | 0.01 | <0.01 | <0.01 | **1** | 0.75 | -0.46 | 0.57 | -0.70 |
| 2 | 0.09 | 0.88 | 0.77 | 0.42 | **2** | 0.30 | 0.03 | 0.05 | -0.15 |
| 3 | 0.02 | 0.89 | 0.78 | 0.72 | **3** | 0.41 | -0.03 | 0.05 | -0.06 |
| 6 | 0.03 | 0.42 | 0.72 | 0.54 | **6** | 0.39 | -0.15 | 0.07 | -0.11 |
| 7 | <0.01 | 0.16 | 0.15 | 0.10 | **7** | 0.72 | -0.26 | 0.26 | -0.30 |
| 11 | 0.54 | 0.01 | <0.01 | <0.01 | **11** | -0.11 | -0.46 | 0.49 | -0.52 |
| 12 | 0.95 | <0.01 | <0.01 | <0.01 | **12** | 0.01 | -0.82 | 0.85 | -0.84 |
| 14 | 0.58 | <0.01 | <0.01 | <0.01 | **14** | 0.10 | -0.72 | 0.72 | -0.75 |
| 16 | 0.18 | 0.14 | 0.02 | <0.01 | **16** | 0.24 | -0.27 | 0.42 | -0.52 |
| 17 | 0.23 | 0.98 | 0.64 | 0.65 | **17** | -0.22 | -0.01 | -0.09 | 0.08 |
| 18 | <0.01 | <0.01 | <0.01 | <0.01 | **18** | -0.81 | -0.83 | 0.78 | -0.65 |
| 19 | 0.07 | 0.08 | 0.11 | 0.26 | **19** | -0.34 | -0.33 | 0.30 | -0.21 |
| 20 | 0.20 | 0.09 | 0.02 | <0.01 | **20** | -0.23 | -0.31 | 0.42 | -0.49 |
| 21 | <0.01 | 0.02 | 0.01 | <0.01 | **21** | 0.65 | -0.42 | 0.48 | -0.60 |
| 22 | 0.93 | 0.38 | 0.35 | 0.24 | **22** | -0.02 | -0.16 | 0.17 | -0.21 |
| 23 | 0.14 | <0.01 | <0.01 | <0.01 | **23** | 0.27 | -0.65 | 0.61 | -0.61 |
| 24 | <0.01 | 0.20 | 0.72 | 0.04 | **24** | 0.71 | 0.23 | -0.07 | -0.36 |
| 25 | 0.26 | <0.01 | <0.01 | <0.01 | **25** | 0.21 | -0.51 | 0.66 | -0.84 |
| 26 | <0.01 | <0.01 | <0.01 | <0.01 | **26** | -0.69 | -0.79 | 0.76 | -0.74 |
| 27 | <0.01 | <0.01 | <0.01 | <0.01 | **27** | 0.60 | -0.54 | 0.55 | -0.55 |
| 28 | 0.05 | <0.01 | <0.01 | <0.01 | **28** | -0.35 | -0.84 | 0.81 | -0.69 |
| 30 | 0.22 | <0.01 | <0.01 | <0.01 | **30** | -0.22 | -0.63 | 0.63 | -0.66 |
| 31 | 0.01 | <0.01 | <0.01 | <0.01 | **31** | -0.44 | -0.87 | 0.86 | -0.78 |
| 32 | 0.40 | 0.90 | 0.85 | 0.69 | **32** | -0.16 | -0.02 | 0.03 | -0.07 |
| 33 | 0.84 | 0.71 | 0.67 | 0.37 | **33** | 0.04 | -0.07 | 0.08 | -0.16 |
|  |  |  |  |  |  |  |  |  |  |
|  |  |  |  |  | **mean** |  | **-0.70** | **+0.68** | **-0.67** |
|  |  |  |  |  | **std** |  | **0.15** | **0.13** | **0.11** |

­­­­­
