# Supplementary material for: Objective differentiation of neonatal EEG background grades using detrended fluctuation analysis
Source: Front Hum Neurosci. 2015 Apr 23;9:189. doi: 10.3389/fnhum.2015.00189 (PMC4407610; doi:10.3389/fnhum.2015.00189)
Supplement: Supplementary file 2 [file DataSheet2.DOCX]

1. **Supplementary Material**

**Appendix A**

Detrended Fluctuation Analysis (DFA), was introduced by (Peng et al., 1994) to quantify long-range temporal correlation with weak stationarity assumptions. However, not all time series can be characterized as monofractal processes and described with a single DFA scaling exponent. In such cases, the crossover time points are observable at different scales, the time series have different working regimes. Hence, instead of a single DFA exponent, the time series can be potentially characterized as an interwoven series of different fractal exponents – multifractal. The outline of both algorithms and their differences are given as described in (Kantelhardt et al., 2002; Hardstone et al., 2012):

*Step 1*: compute the cumulative sum of the time series to create the signal profile *Y:*

$Y\left( i \right)=\sum_{k=1}^{i} {(x}_{k})-\left\langle x \right\rangle,\left\langle x \right\rangle=mean of the time series$, *i*=1,2,…,*N*.

*Step 2:* Define a set of *N_s_*=int(*N/s*) window sizes of length *s*, which are equally spaced on a logarithmic scale between the lower bound of four samples and the maximum window size.

*Step 3*: Calculate the local trend *y_ν_* of each of the segments *N_s_* using a least-squares fit of the series. Next, determine the variance of the detrended segment as a function of window size *s* and the segment number *ν:*

$$F^{2}\left( s,\nu\right)=\frac{1}{s}\sum_{i=1}^{s} \left\{ Y\left[ \left( \nu-1 \right)s+i \right]-y_{\nu}(i) \right\}^{2}, \nu=1,..,N_{s}$$

*Step 4:* Average over all segments to obtain the *q*^th^ order fluctuation function:

$$F_{q}\left( s \right)=\left\{ \frac{1}{N_{s}}\sum_{v=1}^{Ns} \left[ F^{2}(s,v) \right]^{q/2} \right\}^{1/q}$$

For a single value *q*=2, the monofractal DFA algorithm is obtained. The fluctuation function *F* is plotted along the logarithmic axes. The DFA scaling exponent, $\alpha$, is the slope of the linear approximation in the range of time-scales of interest and can be estimated using linear regression.

Alternatively, for multifractal DFA, the value of *q* is commonly selected from -5 till 5, with an equidistant unit step of 0.5 (or 0.2). In this way, a set of *q*-order based fluctuation functions are obtained (e.g. Fig 2B1 and 2B2). Within the MF-DFA paradigm, the large variations will be dominant for positive *q* values, whereas the small fluctuations will be characterized with negative *q* values. In contrast, monofractal DFA will be mainly insensitive to local fluctuations.

**Appendix B**

Automated IBI detector: To detect suppressed periods, EEG channels are independently adaptively segmented with an algorithm proposed in (Krajca et al., 1991). The main goal of the segmentation step is to distinguish and separate locally suppressed EEG periods from burst periods (Fig. B1 – A1). Subsequently, we classify segments into low amplitude class (L) if the segments’ amplitude is approximately below 10µV (Fig. B1 – A2). To detect globally suppressed EEG periods after the segmentation/classification step, we monitor the spatial and temporal distribution of low amplitude segments across EEG channels. To accomplish this, we count the number of EEG channels expressing low amplitude segments at a particular time instant. This results in a new signal, which we call temporal profile (Fig. B1 – A3). Consequently, we define an IBI if the temporal profile signal exceeds half of the total number of EEG channels for more than 3 seconds. Examples of IBI detection are illustrated in Fig. B1.

**Figure B1.**

**
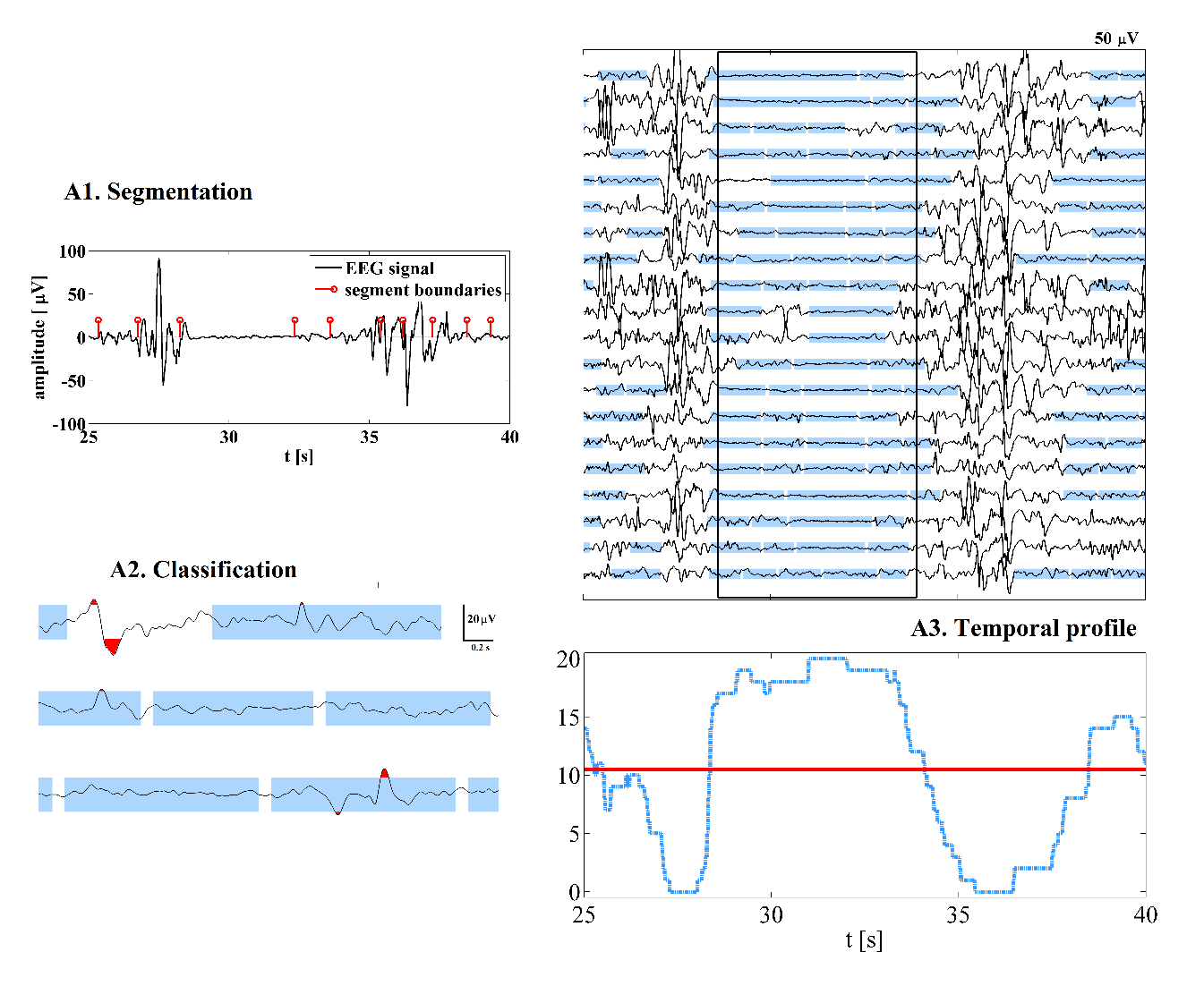
**
